# A Gradient Boosting Algorithm for Survival Analysis via Direct Optimization of Concordance Index

**DOI:** 10.1155/2013/873595

**Published:** 2013-11-20

**Authors:** Yifei Chen, Zhenyu Jia, Dan Mercola, Xiaohui Xie

**Affiliations:** ^1^Department of Computer Science, University of California Irvine, Irvine, CA 92697, USA; ^2^Department of Statistics, The University of Akron, Akron, OH 44325, USA; ^3^Department of Family and Community Medicine, Northeast Ohio Medical University, Rootstown, OH 44272, USA; ^4^Department of Pathology and Laboratory Medicine, University of California Irvine, Irvine, CA 92697, USA; ^5^Institute for Clinical and Translational Cancer Biology, University of California Irvine, Irvine, CA 92697, USA; ^6^Institute for Genomics and Bioinformatics, University of California Irvine, Irvine, CA 92697, USA

## Abstract

Survival analysis focuses on modeling and predicting the time to an event of interest. Many statistical models have been proposed for survival analysis. They often impose strong assumptions on hazard functions, which describe how the risk of an event changes over time depending on covariates associated with each individual. In particular, the prevalent proportional hazards model assumes that covariates are multiplicatively related to the hazard. Here we propose a nonparametric model for survival analysis that does not explicitly assume particular forms of hazard functions. Our nonparametric model utilizes an ensemble of regression trees to determine how the hazard function varies according to the associated covariates. The ensemble model is trained using a gradient boosting method to optimize a smoothed approximation of the concordance index, which is one of the most widely used metrics in survival model performance evaluation. We implemented our model in a software package called GBMCI (gradient boosting machine for concordance index) and benchmarked the performance of our model against other popular survival models with a large-scale breast cancer prognosis dataset. Our experiment shows that GBMCI consistently outperforms other methods based on a number of covariate settings. GBMCI is implemented in R and is freely available online.

## 1. Introduction

Survival analysis focuses on developing diagnostic and prognostic models to analyze the effect of covariates on the outcome of an event of interest, such as death or disease recurrence in disease studies. The analysis is often carried out using regression methods to estimate the relationship between the covariates and the *time to event* variable. In clinical trials, time to events is usually represented by *survival times*, which measure how long a patient with a localized disease is alive or disease-free after treatment, such as surgery or surgery plus adjuvant therapy. The covariates used in predicting survival times often include clinical features, such as age, disease status, and treatment type. More recently, molecular features, such as expression of genes, and genetic features, such as mutations in genes, are increasingly being included in the set of covariates. Survival analysis also has applications in many other fields. For instance, it is often used to model machine failure in mechanical systems. Depending on specific circumstances, survival times may also be referred to as *failure times*.

A major complication for survival analysis is that the survival data are often incomplete due to censoring, because of which standard statistical and machine learning tools on regression cannot be readily applied. The most common type of censoring occurring in clinical trials is right censoring, where the survival time is known to be longer than a certain value but its precise value is unknown. This can be due to multiple reasons. For instance, a patient might withdraw from a clinical trial or a clinical trial might end early such that some patients are not followed up with afterwards.

Many statistical methods have been developed for survival analysis. One major category of these methods adopts a likelihood-based approach. An essential component of the models in this category is the estimation of the hazard function *λ*(*t*), defined as the event rate at time *t* conditional on survival up to time *t*. Different models often impose different assumptions on the forms of the hazard function. In particular, the proportional hazards (PH) model (also called the Cox model), one of the most prevalent models in survival analysis, assumes that different covariates contribute multiplicatively to the hazard function [1–4]. To relax the proportional hazards assumption and allow for more complicated relationships between covariates, parametric models based on artificial neural networks (ANN) [5–8] and ensembles of tree models based on boosting [9–12] have also been proposed. In order to handle the censored data, all these models use an approximation of the likelihood function, called the Cox partial likelihood, to train the predictive model. The partial likelihood function is computationally convenient to use; however, it is unclear how well the full likelihood can be approximated by the partial likelihood.

Many other methods aiming at optimizing a different class of objective functions rather than the partial likelihood have also been proposed. Some of these methods adapt existing regression models to estimate the relationship between survival times and covariates, by taking the censored data into account in training the models [13, 14], while others adopt a classification-based framework and train their models using only the rank information associated with the observed survival times [8, 15, 16]. Recently, random survival forests [17, 18], a new ensemble-of-trees model based upon bagging, became popular in survival analysis. They resort to predicting either the cumulative hazard function or the log-transformed survival time.

In clinical decision making, physicians and researchers are often more interested in evaluating the *relative risk* of a disease between patients with different covariates than the absolute survival times of these patients. For this purpose, Harrell et al. introduced the important concept of *concordance index* (C-index, concordance C, or simply CI) as a measure of the separation between two survival distributions [19, 20]. Given two survival distributions, the C-index computes the fraction of pairs of patients with consistent risk orders over the total number of validly comparable pairs. Because of its focus on assessing the accuracy of relative risk, the C-index is widely adopted in survival model performance evaluation, where the order of predicted survival times is compared to the order of the observed ones [21–23].

Our goal in this paper is to develop a new survival model to capture the relationship between survival times and covariates by directly optimizing the C-index between the predicted and observed survival times. Although both the Cox model based on partial likelihood and the ranked-based methods mentioned above also utilize only the order information between survival times, the C-index based method provides a more principled way of combining all pairwise order information into a single metric. There have been prior attempts in directly learning the C-index for survival analysis, including a neural network based model [21] and an extension of the Cox model trained using a lower bound of C-index [22]. However, both methods impose parametric assumptions on the effect of covariates on survival times. Our contribution here is to adopt a nonparametric approach to model the relationship between survival times and covariates by using an ensemble of trees and to train the ensemble model by learning the C-index.

In the following, we will provide a detailed description of our ensemble survival model based on learning the C-index. We will derive an algorithm to train the model using the gradient boosting method originally proposed by Friedman [9]. The algorithm is implemented in an R software package called GBMCI (gradient boosting machine for concordance index), which is freely available at https://github.com/uci-cbcl/GBMCI. We benchmark the performance of GBMCI using a large-scale breast cancer prognosis dataset and show that GBMCI outperforms several popular survival models, including the Cox PH model, the gradient boosting PH model, and the random survival forest, in a number of covariate settings.

## 2. Materials and Methods

### 2.1. Survival Analysis

We review the basic concepts of survival analysis here. For a systematic treatment, see [24, 25]. In survival analysis, the time to event (death, failure, etc.) *t* is typically modeled as a random variable, which follows some probability density distribution *p*(*t*). The density can be characterized by the *survival function S*(*t*) = Pr(*T* > *t*) = ∫_*t*_^*∞*^ *p*(*T*)d*T* for *t* > 0. The survival function captures the probability that the event does not happen until time *t*. A closely-related concept is the *hazard function λ*(*t*) = lim⁡_Δ*t*→0_(Pr⁡(*t* < *T* < *t* + Δ*t* | *T* > *t*))/Δ*t* = *p*(*t*)/*S*(*t*), which measures the event rate at time *t* conditioned on survival until *t*. One can further show that *S*(*t*) = *e* ^−∫_0_^*t*^*λ*(*τ*)d*τ*^.

The likelihood function for right-censored survival data is expressed as

(1) L(θ;{xi,ti,δi}i=1n)=∏i∈Ep(ti ∣ xi,θ)∏j∈CS(tj ∣ xj,θ)  =∏i=1nλ(ti ∣ xi,θ)δiS(ti ∣ xi,θ).

Note the augmentation of our notation (we will follow this convention in the following context unless otherwise stated): *θ* is the set of regression parameters of the survival/hazard model; *δ*_*i*_,  *i* = 1,…, *n*, indicates whether the event happens (*δ* = 1), or not (*δ* = 0, i.e., the data is censored); *x*_*i*_,  *i* = 1,…, *n*, are the explanatory covariates that affect the survival time; *E* is the set of data whose events are observed; and *C* is the set of censored data. The full maximum-likelihood approach would optimize *L* over the functional space of *S* (or *λ*) and parameter space of *θ*. Unfortunately, this is often intractable.

#### 2.1.1. Proportional Hazard Model

In his seminal work [1, 2], Cox introduced the *proportional hazard* (PH) model *λ*(*t* | *x*, *θ*) = *λ*_0_(*t*)exp⁡{*x* ^*T*^ *θ*}. *λ*_0_(*t*) is the *baseline* hazard function; exp⁡{*x* ^*T*^ *θ*} is the relative hazard, which summarizes the effect of covariates. Cox observed that under the PH assumption, it suffices to estimate *θ* without the necessity of specifying *λ*_0_(*t*) and optimizing the likelihood (1). Instead, he proposed to optimize the so-called Cox partial likelihood

(2) $$\begin{array}{c} L_{p}\left(\theta ;{\left\{x_{i},t_{i},\delta_{i}\right\}}_{i=1}^{n}\right)=\underset{i\in E}{\prod }\frac{\exp\left\{\theta^{T}x_{i}\right\}}{\sum_{j:t_{j}\geq t_{i}}^{}\exp\left\{\theta^{T}x_{j}\right\}}. \end{array}$$

The Cox model has become very popular in evaluating the covariates' effect on survival data and is generalized to handle time-varying covariates and time-varying coefficients [3, 4]. However, the proportional hazards assumption and the maximization of the partial likelihood remain two main limitations. Nonlinear models, for example, multilayer neural networks [5–7], have been proposed to replace *θ* ^*T*^ *x*. However, they still assume parametric forms of the hazard function and attempt to optimize the partial likelihood.

#### 2.1.2. Concordance Index

The *C-index* is a commonly used performance measure of survival models. Intuitively, it is the fraction of all pairs of patients whose predictions have correct orders over the pairs that can be ordered. Formally, the C-index is

(3) CI=1|𝒫|∑(i,j)∈𝒫I(F(xi)<F(xj))=1|𝒫|∑i∈E ∑j:tj>tiI(F(xi)<F(xj)).

*𝒫* is the set of validly orderable pairs, where *t*_*i*_ < *t*_*j*_; |*𝒫*| is the number of pairs in *𝒫*; *F*(*x*) is the prediction of survival time; *I* is the indicator function of whether the condition in parentheses is satisfied or not. In the PH setting, the predicted survival time can be equivalently represented by the negative log relative hazard. The C-index estimates the probability that the order of the predictions of a pair of comparable patients is consistent with their observed survival information.

### 2.2. Gradient Boosting Machine

The *gradient boosting machine* (GBM) is an ensemble learning method, which constructs a predictive model by additive expansion of sequentially fitted weak learners [9, 10]. The general problem is to learn a functional mapping *y* = *F*(*x*; *β*) from data {*x*_*i*_, *y*_*i*_}_*i*=1_^*n*^, where *β* is the set of parameters of *F*, such that some cost function ∑_*i*=1_^*n*^Φ(*y*_*i*_, *F*(*x*_*i*_; *β*)) is minimized. Boosting assumes *F*(*x*) follows an “additive” expansion form *F*(*x*) = ∑_*m*=0_^*M*^ *ρ*_*m*_ *f*(*x*; *τ*_*m*_), where *f* is called the *weak* or *base learner* with a weight *ρ* and a parameter set *τ*. Accordingly, {*ρ*_*m*_, *τ*_*m*_}_*m*=1_^*M*^ compose the whole parameter set *β*. They are learnt in a greedy “stage-wise” process: (1) set an initial estimator *f*_0_(*x*); (2) for each iteration *m* ∈ {1,2,…, *M*}, solve (*ρ*_*m*_, *τ*_*m*_) = argmin⁡_*ρ*,*τ*_∑_*i*=1_^*n*^Φ(*y*_*i*_, *F*_*m*−1_(*x*_*i*_) + *ρf*(*x*_*i*_; *τ*)). GBM approximates (2) with two steps. First, it fits *f*(*x*; *τ*_*m*_) by

(4) $$\begin{array}{c} \tau_{m}=\arg\underset{\tau }{\min}\overset{n}{\underset{i=1}{\sum }}{\left(g_{im}-f(x_{i};\tau )\right)}^{2}, \end{array}$$

where

(5) $$\begin{array}{c} g_{im}=-{\left[\frac{\partial \Phi \left(y_{i},F\left(x_{i}\right)\right)}{\partial F\left(x_{i}\right)}\right]}_{F(x)=F_{m-1}(x)}. \end{array}$$

Second, it learns *ρ* by

(6) $$\begin{array}{c} \rho_{m}=\arg\underset{\rho }{\min}\overset{n}{\underset{i=1}{\sum }}\Phi \left(y_{i},F_{m-1}\left(x_{i}\right)+\rho f\left(x_{i};\tau_{m}\right)\right). \end{array}$$

Then, it updates *F*_*m*_(*x*) = *F*_*m*−1_(*x*) + *ρ*_*m*_ *f*(*x*; *τ*_*m*_). In practice, however, *shrinkage* is often introduced to control overfitting, and the update becomes *F*_*m*_(*x*) = *F*_*m*−1_(*x*) + *νρ*_*m*_ *f*(*x*; *τ*_*m*_), where 0 < *ν* ≤ 1. If the weak learner is the regression tree, the complexity of *f*(*x*) is determined by tree parameters, for example, the tree size (or depth), and the minimum number of samples in terminal nodes. Besides using proper shrinkage and tree parameters, one could improve the GBM performance by *subsampling*, that is, fitting each base learner on a random subset of the training data. This method is called *stochastic gradient boosting* [10].

Compared to parametric models such as *generalized linear models* (GLM) [26] and neural networks, GBM does not assume any functional form of *F* but uses additive expansion to build up the model. This nonparametric approach gives more freedom to researchers. GBM combines predictions from the ensemble of weak learners and so tends to yield more robust results than the single learner. Empirically, it also works better than the bagging-based random forests [27], probably due to its functional optimization motivation. However, it requires the cost function Φ to be differentiable with respect to *F*. GBM has been implemented in the popular open-source R package “gbm” [12] which supports several regression models.

#### 2.2.1. Boosting the Proportional Hazard Model

Ridgeway [11] adapted GBM for the Cox model. The cost function is the negative log partial likelihood:

(7) $$\begin{array}{c} \Phi \left(y,F\right)=-\overset{n}{\underset{i=1}{\sum }}\delta_{i}\left\{F\left(x_{i}\right)-\log\left(\underset{j:t_{j}\geq t_{i}}{\sum }e^{F(x_{j})}\right)\right\}. \end{array}$$

One can then apply (4), (5), and (6) to learn each additive model. In the “gbm” package, this cost function corresponds to the “coxph” distribution and is further optimized to refit terminal nodes with Newton's method. We denote this particular GBM algorithm as GBMCOX and its implementation in the “gbm” package as “gbmcox.”

### 2.3. Concordance Index Learning via Gradient Boosting

We now propose a gradient boosting algorithm to learn the C-index. As the C-index is a widely used metric to evaluate survival models, previous works [21, 22] have investigated the possibility to optimize it, instead of Cox's partial likelihood. However, these works are limited to parametric models, such as linear models or neural networks. Our key contribution is to tackle the problem from a nonparametric ensemble perspective based on gradient boosting.

Optimizing the C-index directly is difficult because of its discrete nature, that is, the summation over indicator functions in (3). We resort to the differentiable approximation proposed in [21], which adopts the logistic sigmoid function in each term. We call it the *smoothed concordance index* (SCI). Specifically,

(8) $$\begin{array}{c} \text{SCI}=\frac{1}{\left|𝒫\right|}\underset{(i,j)\in 𝒫}{\sum }\frac{1}{1+e^{\alpha (F(x_{i})-F(x_{j}))}}, \end{array}$$

where *α* is a hyperparameter that controls the steepness of the sigmoid function (accordingly, the approximability of SCI to CI) and *F*(*x*) is the prediction of survival time. Let Φ(*y*, *F*) = −SCI. Then, at each iteration *m* > 0 of gradient boosting,

(9) gim=[∂SCI∂F(xi)]F(x)=Fm−1(x)=α|𝒫|{∑(k,i)∈𝒫eα(Fm−1(xk)−Fm−1(xi))[1+eα(Fm−1(xk)−Fm−1(xi))]2     −∑(i,j)∈𝒫eα(Fm−1(xi)−Fm−1(xj))[1+eα(Fm−1(xi)−Fm−1(xj))]2}.

So the base learner *f*(*x*; *τ*_*m*_) can be fitted using {*g*_*im*_}_*i*=1_^*n*^ and (4). Next,

(10) $$\begin{array}{c} \rho_{m}=\arg\underset{\rho }{\max}\frac{1}{\left|𝒫\right|}\underset{(i,j)\in 𝒫}{\sum }\frac{1}{1+e^{\alpha (F_{m-1}(x_{i})+\rho f(x_{i};\tau_{m})-F_{m-1}(x_{j})-\rho f(x_{j};\tau_{m}))}}. \end{array}$$

Although differentiable, SCI has a complicated error surface and is neither convex nor concave. This brings two problems. First, the algorithm's performance depends on its initialization which may lead to different local optima; second, it is difficult to find the global solution of *ρ*_*m*_ in (10). In our implementation, we set the initial estimation {*f*_0_(*x*_*i*_)}_*i*=1_^*n*^ as the prediction from a fitted PH model and use line search to detect *ρ*_*m*_ locally. Empirically, we have found that these heuristics work well for the algorithm.

Algorithm 1, named as GBMCI, summarizes our whole algorithm, which also incorporates the stochastic boosting mechanism [10]. Note that ensemble size *M* is an important parameter that requires tuning, as small *M* may not capture the true model, while large *M* makes the algorithm apt to overfitting. In practice, it is often selected by cross validation. We implement GBMCI in the “gbm” package, under a new distribution called “sci,” which shares the same regression tree engine and complete software architecture as “gbmcox” does. We name our implementation of GBMCI as “gbmsci.” 

## 3. Results

### 3.1. Dataset and Feature Extraction

We illustrate the utility of GBMCI on a large breast cancer dataset, which was originally released by Curtis et al. [28]. The dataset was adopted by the Sage Dream Breast Cancer Challenge (BCC) [29], where it was named *Metabric*. It contains gene expressions, copy number variations, clinical information, and survival data of 1,981 breast cancer patients. The gene expression data consist of 49,576 microarray probes; the copy number data consist of 18,538 SNP probes; the clinical data contain 25 clinical covariates; the survival data contain the survival time and status (dead or censored). Following the convention of BCC, we reserve 1001 patients for training and the other 980 for testing. We applied several successful feature selection schemes from the top competitors in BCC. See Table 1 for details on how these features were generated.

### 3.2. Experimental Settings

As a boosting model, GBMCI's main competitor is the boosted proportional hazard model GBMCOX. As they share identical software environment with a common regression tree engine, the comparison should be reliable and reasonable. For baseline evaluation, we investigate the performance of the PH model with a stepwise Akaike information criterion (AIC) model selection scheme (denoted as “cox”). In addition, we also consider the popular random survival forest (RSF) approach by Ishwaran et al. [18], which is implemented in the R package *randomSurvivalForest* [30] (denoted as “rsf”). We use the concordance index as the evaluation criteria. All experiments are performed in R 2.15.1 software environment.

For “gbmsci,” the hyperparameter *α* controls how well SCI approximates CI. Large *α* values make the approximation good, but the gradient can be very large or even ill defined and *vice versa*. In practice, we find *α* = 1 strikes a good balance between approximability and numerical stability. The line-search range is [0,100] along the gradient direction. The shrinkage *ν* in “gbm” is 0.001 by default. In our experiments, we find *ν* = 0.002 works well for “gbmcox” and *ν* = 1 does for “gbmsci.” We do not essentially apply shrinkage for “gbmsci,” because the small line-search range [0,100] does not necessarily detect the global optimal *ρ*, thus it implicitly contributes to shrinkage. This is mainly for computational efficiency purpose. “gbmsci” and “gbmcox” share other important parameter configurations: maximum number of trees is 1500 (actual number is automatically tuned by 5-fold cross validation); tree depth is 6; *n*_*s*_/*n* (see Algorithm 1) is 1 or 0.5. For “rsf,” the number of trees is 1500; other parameters use default configurations.

### 3.3. Empirical Comparison

**Figure 1:**
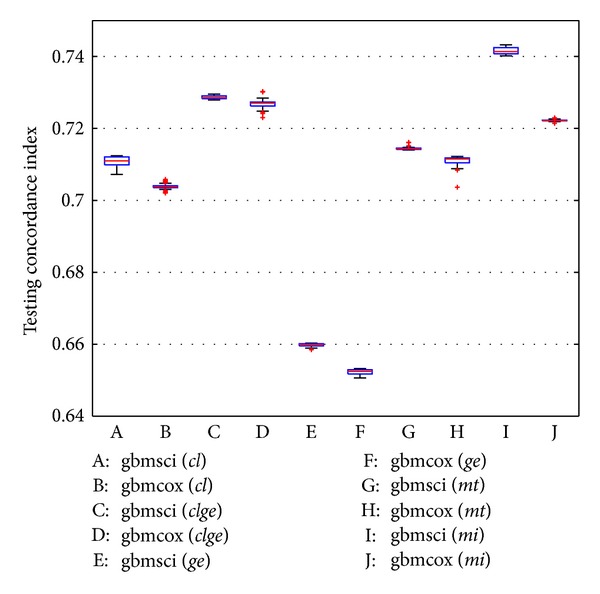
Predictive performance I of GBM methods on the breast cancer dataset. The box plots show the predictive concordance indices of “gbmsci” and “gbmcox” in 50 random experiments without subsampling, using the five feature representations explained in Table 1. In each box plot, the central red line indicates the median C-index; the blue box is the [25%, 75%] area; the black whiskers reach the upper and lower extremes not including outliers; the red “+” symbols represent the outliers.

Each method is tested using the five feature representations in Table 1. For “gbmsci” and “gbmcox”, as cross validation introduces randomness by partitioning the training data, we repeat the experiment 50 times. Their predictive concordance indices are shown in Figures 1 and  S1 (see Figure S1 in Supplementary Material available online at http://dx.doi.org/10.1155/2013/873595). For “cox”, the predictive concordance indices are shown in Table 2, which also summarizes the performances of “gbmsci” and “gbmcox.” For “rsf,” we also do 50 random tests because of bootstrapping when growing trees. The predictive concordance indices are shown in Figure S2.

 Figures 1 and S1 show that “gbmsci” fairly consistently outperforms “gbmcox.” The advantage is notable when using the features of *cl*, *clge*, *ge*, and *mt* (without subsampling) and substantial when using *mi*. “gbmsci” performs slightly worse only when using *mt* (with subsampling) but is still comparable. Further more, all differences except *mt* (with subsampling) are statistically significant (Student's *t*-test, all *P* values <10^−13^). We also note that subsampling generally improves the predictive power of both “gbmsci” and “gbmcox,” except when using *cl*. This is consistent with the theoretical argument of [10, 11].

From Table 2, one can see that “gbmsci” performs better than “cox” overall. The advantage is notable when using *cl* (without subsampling) and substantial when using *clge*, *mt*, and *mi*. For other cases, “gbmsci” and “cox” are comparable. On the other hand, “gbmcox” performs better than or comparable to “cox” for *cl*, *clge*, and *mt* but does slightly worse for *ge* and *mi*. Comparing Figures 1, S1 with S2, one can see that “gbmsci” outperforms “rsf” in most cases, while “gbmcox” also performs better than “rsf” overall.

To summarize the comparative study, GBMSCI outperforms GBMCOX, Cox PH, and RSF in most of the feature-subsampling settings. The results also shed light on the importance of feature representation. First, gene expression data may have potential prognosis power given well-designed feature extraction schemes, for example, the Attractor Metagene (*mt*). Second, combining clinical and gene features together seems to provide enhanced prognosis power over using them separately. This is the case in both the original gene space (*clge*) and the transformed space (*mi*).

## 4. Discussion

Many machine learning techniques have been adapted and developed for survival analysis [23, 32, 33]. In particular, several important parametric models, such as neural networks and support vector regression, have been generalized to handle censored data. They provide survival studies with more comprehensive and flexible methodologies. However, ensemble methods are mostly limited to either direct adaptation of boosting to the classical PH model [11, 12] or bagging approaches such as random survival forests [17, 18]. Our proposed algorithm generalizes the gradient boosting machine to learn the C-index directly, which provides a new ensemble learning methodology for survival analysis. As the C-index is a ranking function in essence [22], our model also serves as an ensemble treatment to the *ranking problem* for survival data. This is novel and has not been addressed previously [14, 34, 35].

By studying the large-scale *Metabric* breast cancer dataset, we found that “gbmsci” overall performs better than “gbmcox,” “cox,” and “rsf” in terms of predictive C-indices. The improvement is notable and consistent when various feature representations were applied. This study also demonstrates the enhanced prognosis power when gene expression profiles and clinical variables are combined and when the gene space is remapped in the predictive model. Interestingly, “gbmsci” typically outperforms “gbmcox” and “cox” when using these informative features. This may provide useful clues for clinical decision making. Moreover, we also confirm the utility of the subsampling scheme of gradient boosting.

Although GBMCI has free parameters that require tuning, for example, *α* and the line-search range, they empirically work well among different experiments once they have been well tuned. In addition, the algorithm still renders similar performance, when *α* is within a reasonable neighborhood of 1 (e.g., *α* = 2). One possible reason for the robustness is that both the objective function (8) and the gradient (9) are upper- and lower-bounded (as can be shown through basic algebraic manipulations). Such bounds are not typically available when optimizing other objective functions for different regression problems, such as the partial likelihood for the Cox model, the mean absolute error for the Lasso regression, and the polynomial alternative of SCI as proposed by [21].

The proposed algorithm has room for improvement. First, current initialization and line-search steps, although working well in practice, are not necessarily the globally optimal strategy. For initialization, one potential alternative is to fit PH models by subsampling or bootstrapping of the training data. To better address the problems, one may have to design other initialization heuristics or adopt a global optimization technique such as Monte Carlo methods. Second, GBMCI is computationally more intensive than other methods, because of the pairwise sigmoid computation in (9) and (10). Fortunately, GBMCI is easily parallelizable, which should help in dealing with large datasets. Third, biomedical research often deals with high-throughput data, for example, microarray gene expression profiling and next generation sequencing data, which require feature selection and dimension reduction. GBMCI does not tackle this task yet. However, as node-splittings of regression trees implicitly perform feature extraction, one could either run GBMCI several iterations and preselect informative variables as a “warm-up” step before the main learning routine or start GBMCI with all variables, iteratively rank their node-split frequencies, and refine the variable pool. These would allow GBMCI perform feature selection and concordance index learning in a unified framework.

Last but not least, we note that ensemble methods are in general more expensive than the Cox model, because of the necessity of tuning parameters, training ensemble weak learners, and cross validation. The tradeoff between predictive power and computational cost remains a question that depends on the specific case requirement. For example, given a particular prognosis analysis task, the Cox model may provide a quick baseline evaluation; ensemble methods could be applied, if higher predictive accuracy and more thorough investigation of covariates' effect are required.

## 5. Conclusion

To summarize, we have developed a new algorithm (GBMCI) for survival analysis. It performs concordance index learning nonparametrically within the gradient boosting framework. It does not impose parametric assumptions on hazard functions, and it expands the ensemble learning methodologies of survival analysis. We implemented GBMCI in an open-source R package, and tested it using a comprehensive cancer prognosis study. GBMCI consistently performs better than three state-of-the-art survival models (the Cox PH model, its boosting expansion, and the random survival forest) over several feature representations. This study also illustrates the importance of feature engineering of clinical and gene expression data in cancer prognosis studies.

## Supplementary Material

The supplementary material consists of two figures, which summarize the predictive performances of GBM-based methods (with subsampling) and the RSF-based method.

## Figures and Tables

**Algorithm 1:**
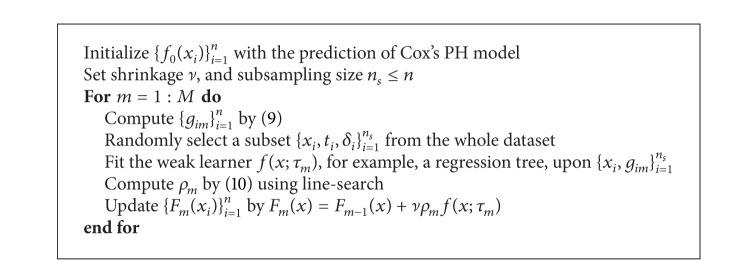
(Stochastic) gradient boosting machine for concordance index learning (GBMCI).

**Table 1:** The five sets of features extracted from the Metabric breast cancer dataset.

Category	Abbreviation	Explanation
Clinical feature	*cl *	A subset of clinical covariates is selected by fitting the Cox model with AIC in a stepwise algorithm. The frequently selected features include age at diagnosis, lymph node status, treatment type, tumor size, tumor group, and tumor grade.
Gene feature	*ge *	A subset of gene expression microarray probes using Illumina HT 12v3 platform is selected whose concordance indices to the survival data are ranked highest (positive concordant) or lowest (negative concordant). A few examples are, “ILMN_1683450,” “ILMN_2392472,” “ILMN_1700337.”
Clinical and gene feature	*clge *	A combination of previously selected clinical features and gene expression features is used to fit the Cox model with AIC in a stepwise algorithm, yielding a refined subset of features.
Metagene feature	*mt *	The high-dimensional gene expression data is fed into an iterative *attractor finding* algorithm, yielding a few Attractor Metagenes which are found commonly present in multiple cancer types [31]. Some multicancer attractors are strongly associated with the tumor stage, grade, or the lymphocyte status.
Clinical and Metagene feature	*mi *	A minimum subset of metagenes which has strong prognosis power for breast cancer [31], combined with several important clinical covariates, such as age at diagnosis and treatment type.

**Table 2:** Numerical statistics of predictive concordance indices of GBM models and the Cox model on the breast cancer dataset. The five feature representations are explained in Table 1. “gbmsci”-I and “gbmcox”-I run without subsampling (*n*_*s*_/*n* = 1), while “gbmsci”-II and “gbmcox”-II run with subsampling (*n*_*s*_/*n* = 0.5). The numerics in each entry show the average C-index and the standard deviation (in parentheses) over 50 random runs. The best performance in each column is highlighted by the bold font.

Model	Feature Representation				
	*cl *	*clge *	*ge *	*mt *	*mi *
“gbmsci”-I	**0.7107** (0.0015)	0.7287 (0.0005)	0.6599 (0.0004)	0.7145 (0.0004)	**0.7416** (0.0010)
“gbmcox”-I	0.7039 (0.0008)	0.7268 (0.0013)	0.6523 (0.0007)	0.7110 (0.0014)	0.7222 (0.0003)
“gbmsci”-II	0.7063 (0.0011)	**0.7341** (0.0014)	**0.6617** (0.0020)	0.7169 (0.0017)	0.7405 (0.0015)
“gbmcox”-II	0.6983 (0.0009)	0.7298 (0.0008)	0.6549 (0.0014)	**0.7173** (0.0010)	0.7306 (0.0008)
“cox”	0.7042	0.7140	0.6590	0.6659	0.7299
