# Supplementary material for: A Gradient Boosting Algorithm for Survival Analysis via Direct Optimization of Concordance Index
Source: Comput Math Methods Med. 2013 Nov 20;2013:873595. doi: 10.1155/2013/873595 (PMC3853154; doi:10.1155/2013/873595)
Supplement: Supplementary file 1 — The supplementary material consists of two figures, which summarize the predictive performances of GBM-based methods (with subsampling) and the RSF-based method. [file 873595.f1.pdf]

Supplimentary Materials

1 Supplimentary Figures

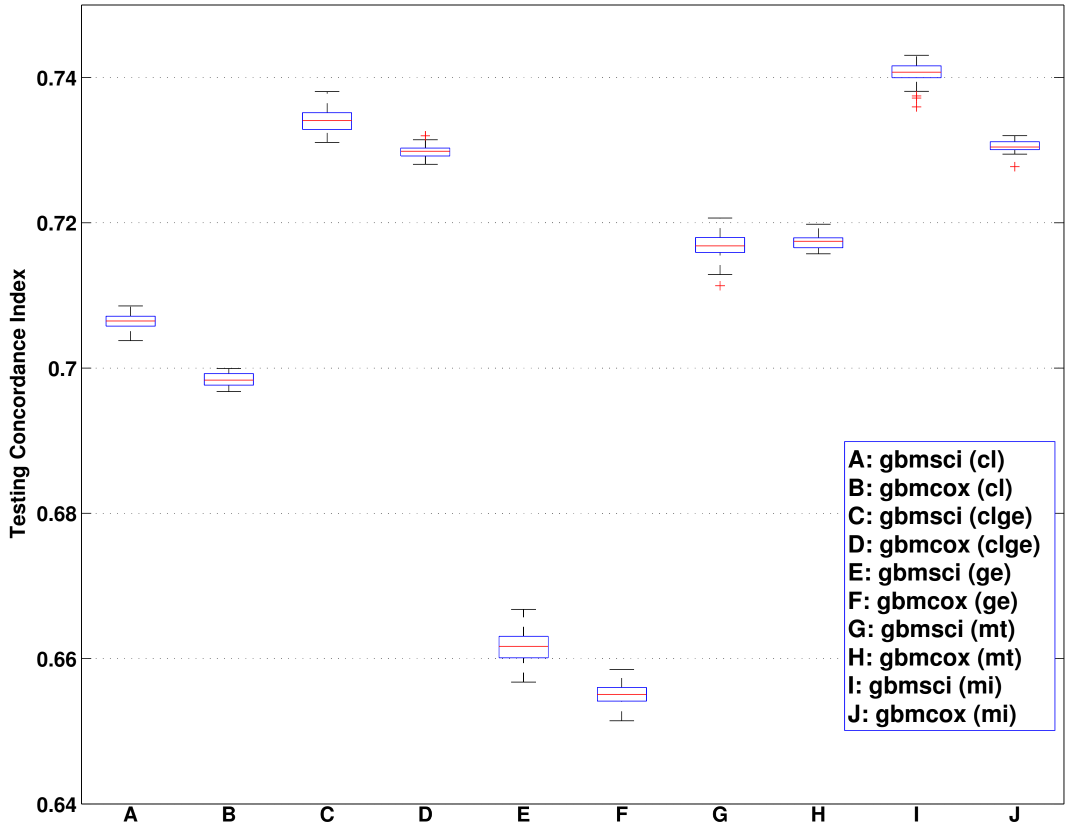

Figure S 1: Predictive performance II of GBM methods on the breast cancer dataset. The box plots show the predictive concordance indices of "gbmsci" and "gbmcox" in 50 random experiments with subsampling ($\frac{n_s}{n} = 0.5$), using the five feature representations explained in Table 1. In each box plot, the central red line indicates the median C-index; the blue box is the $[25\%, 75\%]$ area; the black whiskers reach the upper and lower extremes not including outliers; the red "+" symbols represent the outliers.

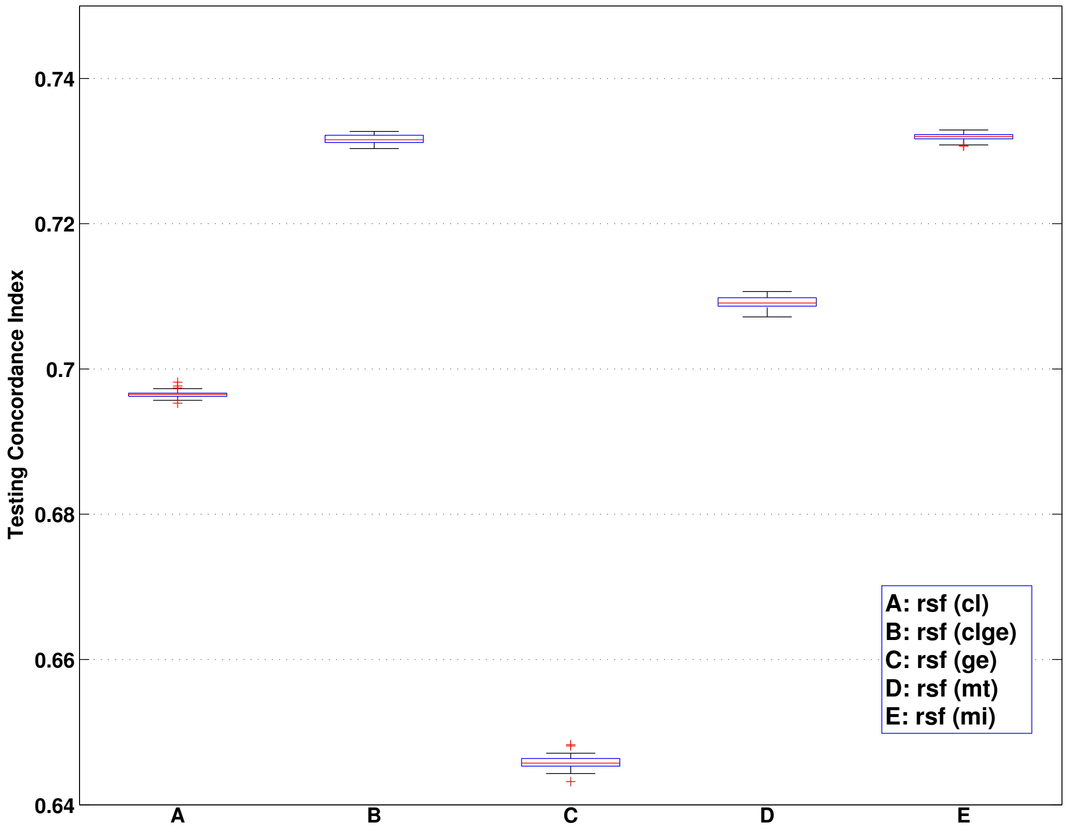

Figure S 2: Predictive performance of the RSF method on the breast cancer dataset. The box plots show the predictive concordance indices of “rsf” in 50 random experiments, using the five feature representations explained in Table 1. In each box plot, the central red line indicates the median C-index; the blue box is the $[25\%, 75\%]$ area; the black whiskers reach the upper and lower extremes not including outliers; the red “+” symbols represent the outliers.
